# Whispering Gallery Mode Resonators for Precision Temperature Metrology Applications

**DOI:** 10.3390/s21082844

**Published:** 2021-04-17

**Authors:** Giovanni Gugliandolo, Shahin Tabandeh, Lucia Rosso, Denis Smorgon, Vito Fernicola

**Affiliations:** 1 Department of MIFT, University of Messina, 98166 Messina, Italy; 2 VTT Technical Research Centre of Finland Ltd., National Metrology Institute VTT MIKES, P.O. Box 1000, FI-02044 VTT, 02150 Espoo, Finland; shahin.tabandeh@vtt.fi; 3 Istituto Nazionale di Ricerca Metrologica, 10135 Torino, Italy; l.rosso@inrim.it (L.R.); d.smorgon@inrim.it (D.S.); v.fernicola@inrim.it (V.F.)

**Keywords:** microwave resonators, whispering gallery modes, metrology, thermometry, temperature measurements

## Abstract

In this work, the authors exploited the whispering gallery mode (WGM) resonator properties as a thermometer. The sensor is made of a cylindrical sapphire microwave resonator in the center of a gold-plated copper cavity. Two coaxial cables act as antennas and excite the WGM standing waves in the cylindrical sapphire at selected resonance frequencies in the microwave range. The system affords a high quality factor that enables temperature measurements with a resolution better than 15 µK and a measurement standard uncertainty of 1.2 mK, a value approximately three times better than that achieved in previous works. The developed sensor could be a promising alternative to platinum resistance thermometers, both as a transfer standard in industrial applications and as an interpolating instrument for the dissemination of the kelvin.

## 1. Introduction

During the last decade, whispering gallery mode (WGM) resonators have attracted considerable attention in sensing applications because of their high sensitivity. They were employed as gas sensors, biosensors and for pressure and force measurements [1,2,3,4,5,6,7,8]. In this article, a sapphire-based whispering gallery mode resonator is employed as a thermometer with promising results in terms of sensitivity, resolution and accuracy.

A sapphire whispering gallery thermometer (SWGT) was described for the first time by Strouse in 2007 [9]. He used a sapphire disk as a dielectric resonator for whispering gallery mode support. The author related the resonance frequency shift to a temperature variation and, after calibration, he was able to measure temperature from 0 to 100 °C with a measurement uncertainty of 10 mK [9]. Five different WGMs were investigated in the band from 14 to 20 GHz, showing a quality factor (*Q*) ranging from 20,000 to 90,000. According to that study, the temperature dependence of the resonant frequency comes from the thermal coefficient of the sapphire dielectric constant and the disk thermal expansion. This dependence can be expressed as follows [9,10]:

(1) $$\frac{1}{f_{0}}\frac{\partial f_{0}}{\partial T}=\frac{1}{f_{0}}\left(\frac{\partial f_{0}}{\partial \varepsilon_{⏊}}\frac{\partial \varepsilon_{⏊}}{\partial T}+\frac{\partial f_{0}}{\partial \varepsilon_{\|}}\frac{\partial \varepsilon_{\|}}{\partial T}+\frac{\partial f_{0}}{\partial L}\frac{\partial L}{\partial T}+\frac{\partial f_{0}}{\partial a}\frac{\partial a}{\partial T}\right),$$

where:

- *f*_0_ is the resonance frequency;
- *T* is the temperature;
- *ε*_⏊_ and *ε*_‖_ are the sapphire permittivity in the radial and axial direction respectively;
- *L* is the sapphire disk height;
- *a* is the disk diameter.

The quantity $1/f_{0}\cdot \partial f_{0}/\partial T$ is the fractional frequency change, which depends on the sapphire permittivity and the resonator size (both are temperature dependent). The second contribution is at least one order of magnitude lower than the first one, so that it is usually neglected [11,12,13,14]. For a sapphire WGM resonator, the fractional frequency change was estimated to be 40–70 ppm/K [9,11,12,14].

In 2012 Yu improved the Strouse’s work [11] by developing the spherical-sapphire-based WGM resonator. In that case, a 12-mm spherical sapphire was employed as dielectric material instead of a dielectric disk. The whispering gallery mode at 13.6 GHz was used for temperature detection in the range from −40 to 85 °C. With that prototype, a *Q* factor of 82,000 was achieved and the device was able to measure temperature with an uncertainty of better than 4 mK.

It was suggested in the literature [9] the SWGT could be a good alternative to the standard platinum resistance thermometer (SPRT) because of its high time stability and potentially small uncertainty in temperature measurements. However, the main limitation of such devices is represented by their mechanical instability [15], e.g., if the dielectric resonator is subject to mechanical vibrations or stress due to thermal expansion, the resonator symmetry is broken and the resonant frequency is compromised. This issue affects both the measurement uncertainty and the calibration curve, often requiring a new calibration of the device. This limitation has reduced considerably the SWGT application in metrology area.

In this article the authors report a new design for the SWGT, particularly to improve the mechanical stability of the device and consequently its measurement uncertainty. A 17.6-mm cylindrical sapphire was employed as the dielectric resonator. Five whispering gallery modes were investigated in the frequency range from 6 to 14 GHz with a *Q* factor ranging from 40,000 to 250,000. Measurements were carried out starting from 233 up to 303 K. By using the novel cavity design described in this article, no mechanical instabilities were recorded, thus achieving a measurement standard uncertainty of 1.2 mK and a temperature resolution of 14 μK. Such a feature enables the SWGT for accurate metrology applications like calibrations. It can be also suitable as interpolating instrument for the dissemination of the kelvin.

Moreover, the developed device can be potentially used as a transfer standard in industrial applications replacing the classic industry-standard PRT in all the areas in which a measurement uncertainty below 10 mK is needed (e.g., aerospace, chemical industry and remote weather stations). The cost of the microwave thermometer is comparable with that of a high level PRT (i.e., 1–2 k€), however, with a large scale production it could be significantly reduced [13].

In Section 2 the new SWGT design is reported, with a description of the experimental setup. In Section 3, the experimental measurements on the fabricated SWGT are shown together with a detailed analysis on the uncertainty budget. Finally, in Section 4, the results are discussed and some ideas on future works are given.

## 2. Materials and Methods

### 2.1. Sapphire Whispering Gallery Mode Resonator

**Figure 1:**
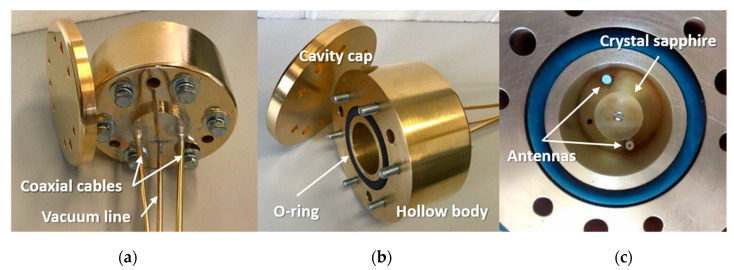
SWGT prototype fabricated. The three panels in the figure report different views of the device: (**a**) top part of the cavity with the two coaxial cables brazed and the vacuum line; (**b**) bottom part of the cavity without the cap, the sealing O-ring and six bolts are visible) and (**c**) 17.6-mm crystal sapphire placed inside the cavity (external diameter = 100 mm) with the exciting antennas.

The SWGT described in this work is made by an equilateral cylindrical resonator: a monocrystalline anisotropic sapphire fabricated by Crystal Systems with the c-axis aligned in the z-direction (±0.1°). Its diameter and height are both 17.6 mm ± 0.05 mm. The crystal surface finish on the outside diameter and the ends is 80/50 according to the standard MIL–13830 [16]. A 2-mm hole along the sapphire *z*-axis enables the positioning of the cylinder inside a gold-plated copper cavity by means of a brass bolt and suitable spacers. The cavity dimensions were designed according to Yu’s studies [17]. The cylindrical cavity should be large enough to avoid losses through the cavity walls and, at the same time, small enough in order to avoid spurious modes inside the cavity. The final dimensions are 35.2 mm for both inner height and diameter. In previous works [9,11,13,18,19] the copper cavity for the SWGT was made of three parts: a central body and two disks, assembled together thus creating a cylindrical metal cavity. In order to improve the mechanical stability, which represents the main issue of such devices, and avoid unwanted displacements of the sapphire due to vibrations and mechanical stress due to thermal expansion, we designed a cavity made by two just parts: a hollow body and a cap (see Figure 1). The body has a blind M2 threaded hole for sapphire positioning, a 3-mm tubing for the connection to a vacuum system and two coaxial cables whose inner connectors protrude inside the cavity acting as antennas. The hollow body and the metal cap are joined together through six M6 bolts, nuts and washers. A Viton^®^ O-ring ensures the inner cavity is sealed with respect to the external environment. The metal cavity is connected to a vacuum system to prevent any water vapor condensation at low temperatures and to minimize the pressure dependency of the resonant frequency. The use of just two parts in the cavity design, with a single O-ring, reduces the risk of leakages. The sapphire assembly stability was further improved by using a longer bolt and a deeper thread inside the body cavity. Six through M6 bolts ensures a better vacuum sealing.

As depicted in Figure 1, three additional 7-mm diameter holes are designed to host SPRTs for experimental characterization and comparison calibration of the SWGT. The coaxial cables were tin brazed into the hollow body at one end and terminated with SMA connectors at the other end. Before the connectors soldering, the coaxial cables were vacuum sealed with an epoxy in order to avoid leakages through the cables. The entire metal block was gold plated with a gold coating thickness larger than 10 µm.

**Figure 2:**
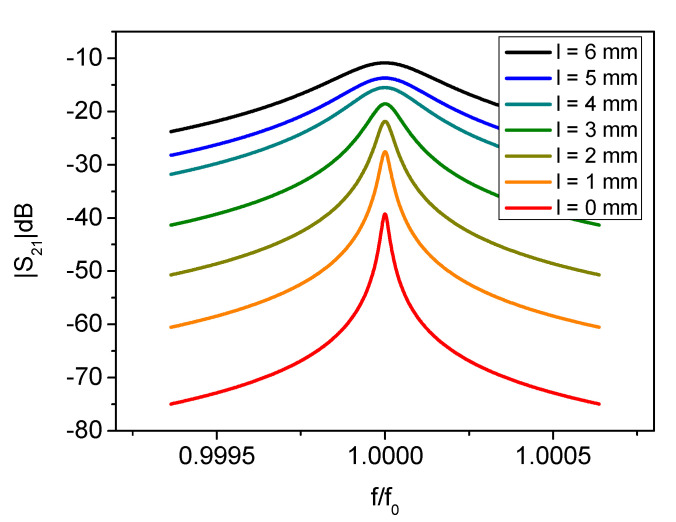
Magnitude of the resonator transmission coefficient (S_21_) with different antennas length (*l*). Only the fitted Lorentzian function of the measured spectrum is reported here. The shorter are the antennas, the higher is the quality factor and the lower is the peak amplitude.

**Figure 3:**
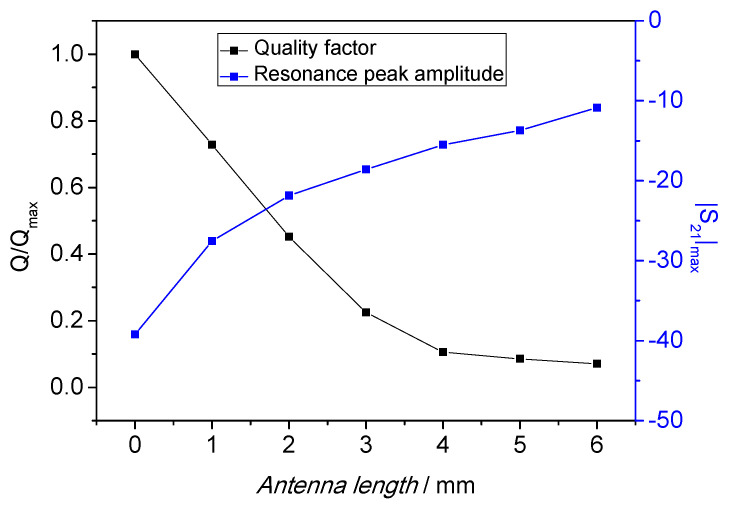
Quality factor and peak amplitude as a function of the antennas length (*l*). In the final design two 0.5-mm long antennas have been fabricated as a compromise between these two quantities.

In order to increase the *Q* factor, computer simulations suggested that the antenna length (*l*) should be minimized. It is very desirable to have a high Q factor since it allows improving the temperature measurement resolution. With long antennas (up to 20 mm), the coupling with the sapphire crystal was very good: it resulted in a high-amplitude resonant peak but with comparatively large bandwidth (i.e., a low *Q* factor). When the antenna length was reduced, as expected, the coupling lowered while the resonant peak was sharper (i.e., a higher *Q* factor) at the cost of a lower signal strength. This can be traced back to a reduction of the coupling between the resonant mode with the neighbors [20]. After the sapphire mounting, the antenna length was trimmed to optimize the system performance. In Figure 2, the measured resonator spectrum (|S_21_|dB) was plotted with different antenna lengths in steps from 6 to 0 mm. For antennas longer than 6 mm, the *Q* factor was too low to be meaningful for this sensing application and the acquired spectrum not reported. The change in *Q* factor and peak amplitude with the antenna length is summarized in Figure 3. As confirmed by numerical simulations, for length of 4 mm and above, the *Q* factor was below 20% of its maximum. When the antenna length was further reduced, the *Q* factor increased inversely with its length. On the other hand, very short antennas make the detection of the resonant peak very challenging because of its low amplitude. In this case, the peak will be affected by noise thus making the estimation of the resonator parameters (e.g., *Q* factor and resonant frequency) difficult. In the final SWGT set up 0.5-mm long antennas were chosen as an acceptable compromise between the *Q* factor and peak amplitude.

### 2.2. Computer Simulations

Five different whispering gallery modes were explored in the frequency range from 6 to 14 GHz. As reported in the literature [21], a WGM can be identified by three integers: *l*, *n* and *m*. They represent the radial, azimuthal and polar components respectively. The integer *l* stands for the number of electromagnetic field maxima along the radial direction of the resonator; the value *2n* represents the number of field maxima in the equatorial plane; and (*n* − *m* + *l)* is the number of field maxima in the polar direction.

The identification of such modes in the resonator spectrum was supported by 3-D EM (electromagnetic) simulations performed during the design/optimization procedure. To this purpose, the device cavity was modeled as a vacuum cylinder (ε_r_ = 1, µ_r_ = 1) with copper as boundary condition (electric conductivity = 5.8 × 10^7^ S/m). The sapphire disk was represented by a solid cylinder having the same dimensions of that used in the experiments and made of an anisotropic material with a permittivity of ε_r_ = 9.391 along the *x* and *y* axis, and ε_r_ = 11.5869 along the *z* axis [11]. Losses in sapphire have been taken into account considering a loss tangent (tan δ) of 1.45 × 10^−5^ in the *x* and *y*-axis and tan δ = 6.05 × 10^−6^ along the *z*-axis [22]. Similarly, the excitation ports were designed into the computer code by referring to the actual size and used materials of the coaxial cables and antennas.

**Figure 4:**
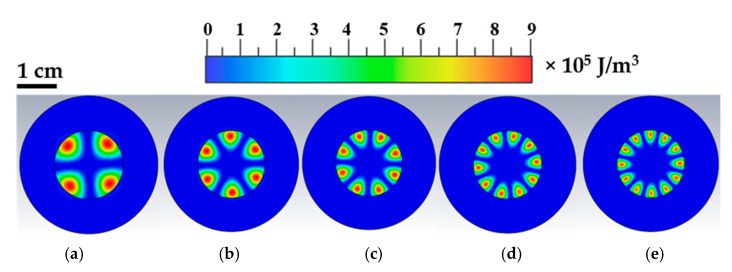
Computer electromagnetic simulations: electric energy densities at resonance. In the frequency range from 6 to 14 GHz, five WGM resonances have been found: (**a**) WGM_n=2_; (**b**) WGM_n=3_; (**c**) WGM_n=4_; (**d**) WGM_n=5_ and (**e**) WGM_n=6_. The five panels report the expected electromagnetic field distribution at the WGM resonances.

A very good agreement between EM simulations and measurements was observed in the resonant frequencies (better than 0.1% of the value) for all the WGMs in the investigated frequency range; for higher azimuthal numbers, the estimated *Q* factor agreed well with the experiments (better than 10%) while for lower *n* the agreement was not satisfactory. This is probably due to an underestimation of the sapphire losses at lower frequencies. Table 1 reports a comparison of the findings. Figure 4, which depicts the electric energy densities at the resonant frequency, shows the expected distribution of whispering gallery modes with the electric field confined in regions close to the external surface of the dielectric resonator. From the number of e-field maxima, the order (*n*) of the WGM can be inferred.

### 2.3. Measurement Setup

**Figure 5:**
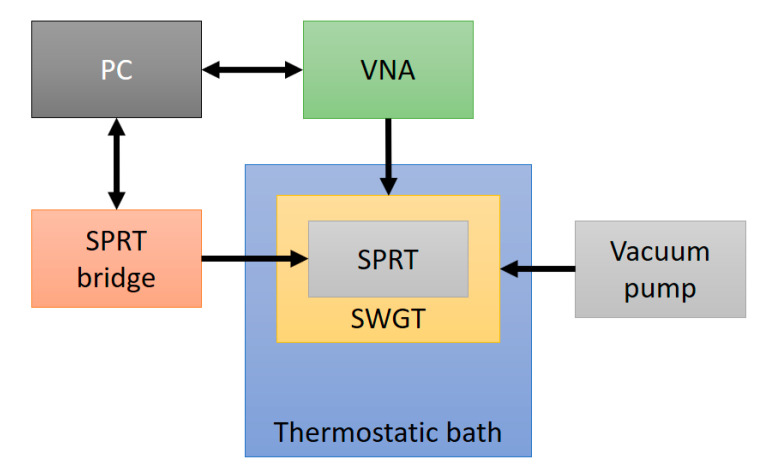
Schematic of the measurement setup. The SWGT was hosted in a thermostatic bath and the microwave resonator was connected to the vacuum pump. The WGM resonant frequencies were detected by a VNA while a SWGT reference temperature was measured by a SPRT and a precision thermometry bridge. Both the VNA and the resistance bridge were controlled by a PC.

The SWGT was tested at 48 different temperatures from −40 to 30 °C, where the upper and lower limit were set by the working temperature of the coaxial cables (from −40 to 125 °C) and the Viton^®^ O-ring gasket (from −45 to 200 °C). It is possible to extend the working temperature range by using metallic O-rings and high temperature range cables or waveguides. The SWGT copper cylinder was hosted inside a Fluke 7100 thermostatic bath and freely suspended in the working volume in order to minimize any mechanical vibrations that could have affected the measurements. The cavity was connected to a vacuum pump and keep running for the entire measurement process in order to guarantee an internal absolute pressure of 1.6 mbar ± 0.1 mbar. A calibrated SPRT, traceable to ITS-90, was inserted in one of the three holes drilled in the copper cavity and its resistance was recorded through the ASL F900 precision thermometry bridge. Finally, a vector network analyzer (VNA) Rhode and Schwartz model ZNB20 was used to record the resonator transmission coefficient (S_21_) at five different frequency bands encompassing the five WGM resonances. In Figure 5 a schematic of the measurement setup is reported.

Measurements made by ASL F900 and R&S ZNB20 were acquired and recorded by a PC. A software code in Python was written for: (a) interfacing and data acquisition of the SPRT resistance from the thermometry bridge and conversion in temperature unit by using the ITS-90 coefficients of the SPRT calibration certificate and (b) interfacing and data acquisition of the transmission coefficient S_21_ from the ZNB20 VNA and Lorentzian fitting of the acquired frequency spectrum for a proper identification of resonant frequency and *Q* factor of the peak [23,24,25]. The fitting algorithm employed was based on the Levenberg–Marquardt method and used a Lorentzian function (*L*(*f*)) in the form [13,26]:

(2) $$L\left(f\right)=\frac{a_{0}f}{f^{2}-f_{0}^{2}},$$

where *a*_0_ and *f*_0_ are two complex quantities estimated by the above best-fit algorithm; *f*_0_ can be written in the form:

(3) $$f_{0}=f_{r}+jg,$$

where *f_r_* is the resonance frequency and *g* the half power bandwidth of the peak. The quality factor can be written as a function of *g* and *f_r_*:

(4) $$Q=\frac{f_{r}/g}{2}.$$

Finally, a complex function *B*(*f*) was used to model the background signal to improve the goodness of the fit [13,26]:

(5) $$B\left(f\right)=\overset{N}{\underset{n=0}{\sum }}b_{n}{\left(f-f_{r}\right)}^{n},$$

where *b_n_* are complex coefficients. Experimental measurements showed that for the present SWGT prototype, it was possible to have an excellent description of the background signal with *n* = 4. The following complex function *T*(*f*) was used for the description of the transmission coefficient:

(6) $$T\left(f\right)=\frac{a_{0}f}{f^{2}-{\left(f_{r}+jg\right)}^{2}}+b_{0}+b_{1}\left(f-f_{r}\right)+b_{2}{\left(f-f_{r}\right)}^{2}+b_{3}{\left(f-f_{r}\right)}^{3}+b_{4}{\left(f-f_{r}\right)}^{4}.$$

It included eight parameters to be calculated by the best-fit algorithm. The software performs the fitting process in real time tracking the resonant frequency, the *Q* factor, the SPRT resistance and converting it into the reference temperature.

**Figure 6:**
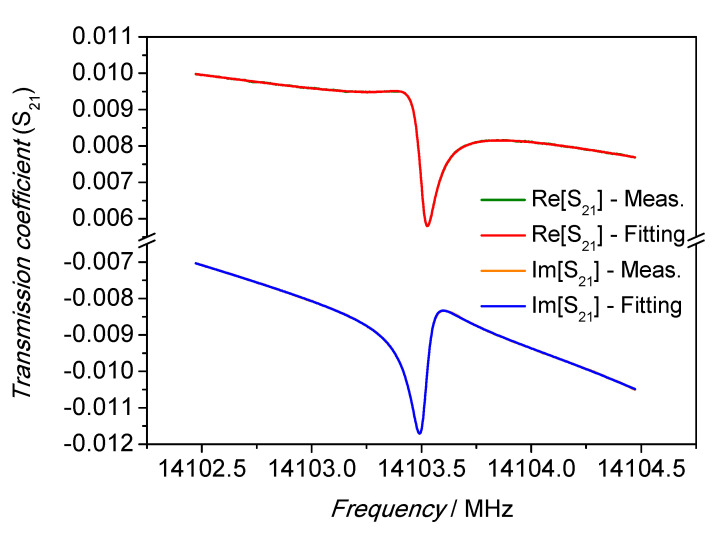
Lorentzian fitting of the frequency spectrum: real and imaginary part. The spectrum was acquired with a resolution of 2.5 kHz. In both cases the fitted functions are perfectly superimposed to the acquired data points.

**Figure 7:**
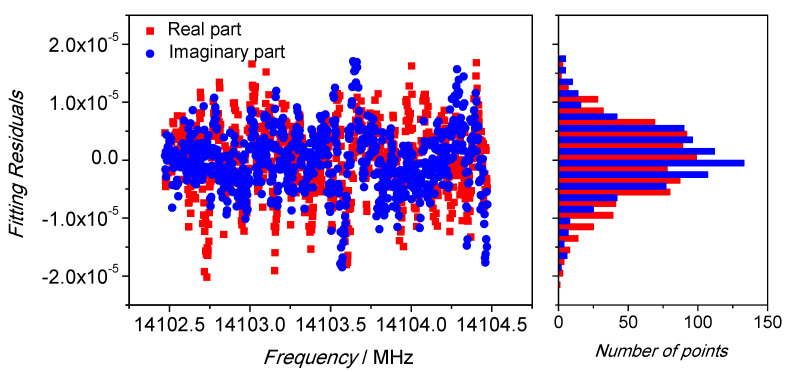
Residuals of the Lorentzian fitting. The scatter is within ±2 × 10^−5^ of the S_21_ transmission coefficient scale; their probability distribution function is normal as a proof of a good mathematical modeling of the resonance.

The performance of the fitting algorithm is showed in Figure 6 for the real and imaginary parts. The fitted functions were perfectly superimposed to the real and imaginary part of the acquired spectrum. The fitting residuals, as reported in Figure 7 for both the real and imaginary part, show their magnitude was below ±2 × 10^−5^ of the transmission coefficient scale, or approximately two order of magnitude lower than the spectrum amplitude. 

## 3. Results

### 3.1. SWGT Calibration

**Figure 8:**
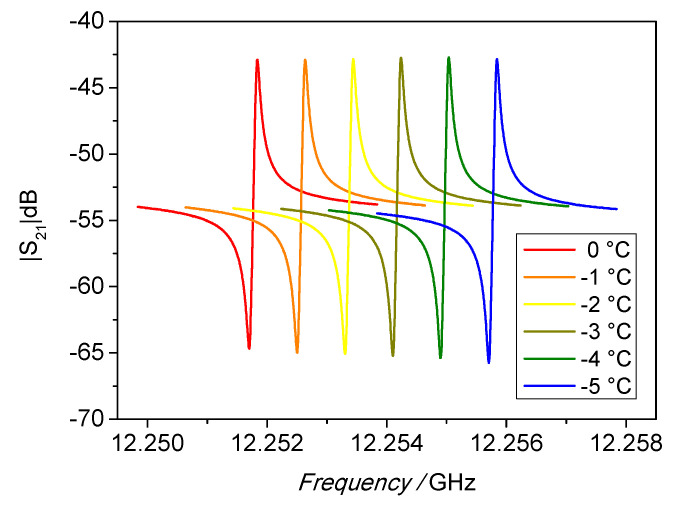
Change of the resonator spectrum with temperature.

**Figure 9:**
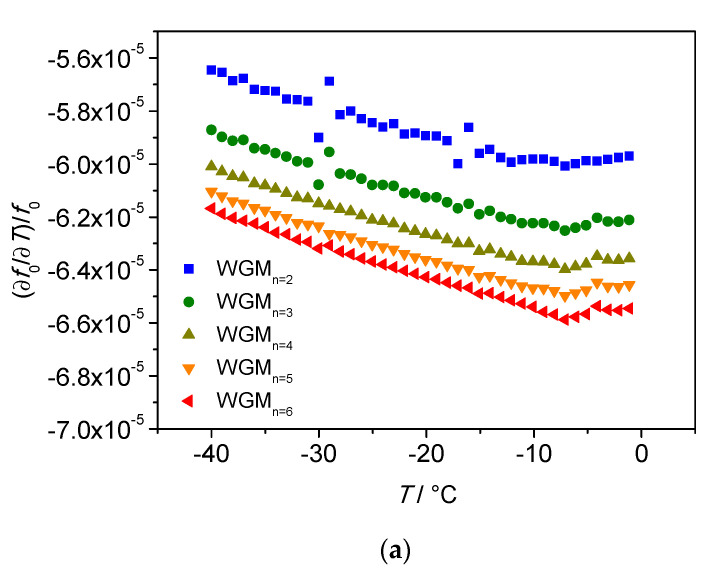

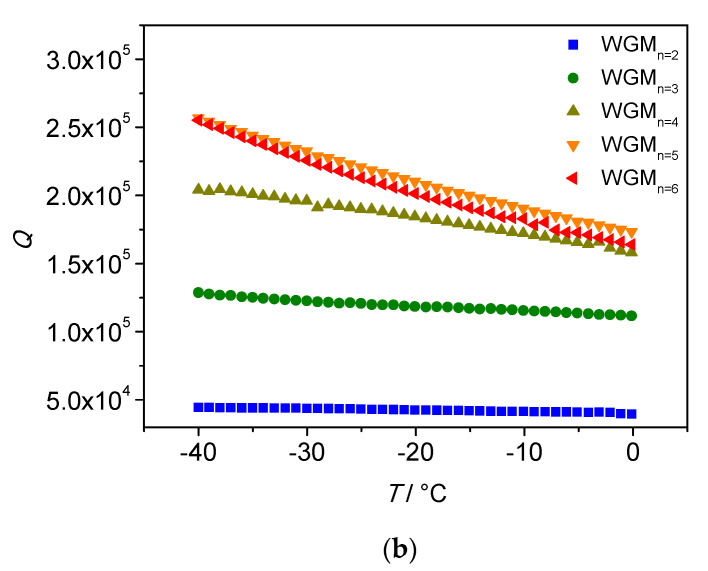
Measurements at five WGM resonances in the temperature range from −40 to 0 °C. (**a**) Frequency fractional change of the developed prototype as a function of the temperature. Small oscillations have been observed in WGM_n=2_ and WGM_n=3_ probably caused by issues in the temperature bath control system. (**b**) *Q* factor change with the temperature.

The SWGT calibration was carried out in the temperature range between −40 and 30 °C. The bath set point temperature was changed approximately every 4.5 h, the first two hours were used for temperature stabilization, the remaining 2.5 h were used to record and fit the spectra of all WGM resonances in the frequency range from 6 (WGM_n=2_) to 14 GHz (WGM_n=6_). For each resonance, a total of one hundred measurements were acquired in a period of approximately 30 min. The change of the resonant peak at 12 GHz with temperature is depicted in Figure 8. The fractional frequency change ($1/f_{0}\cdot \partial f_{0}/\partial T$) is reported in Figure 9a as a function of the temperature, while Figure 9b shows the *Q* factors for the investigated WGMs.

**Figure 10:**
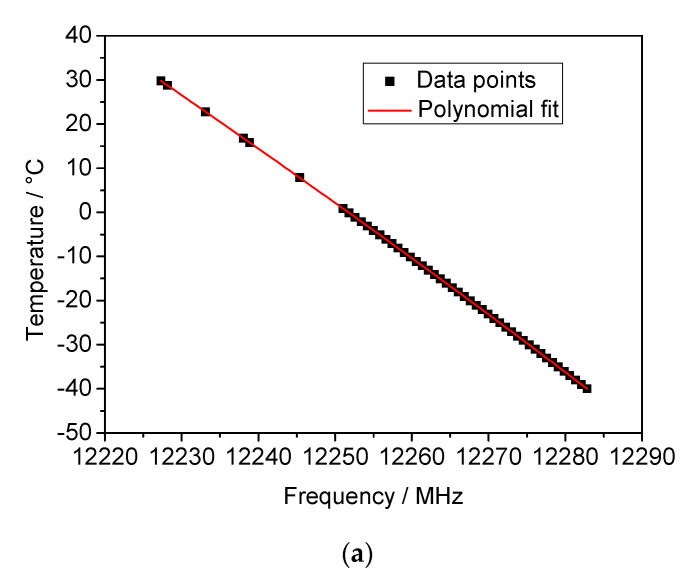

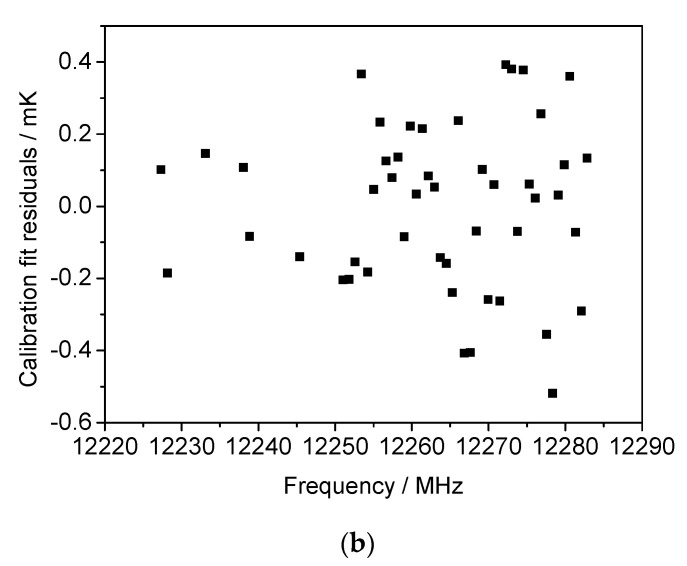
(**a**) Resonant frequency for WGM_n=5_ as a function of the temperature: a 5th order polynomial function (red line) was used to fit the experimental data points. (**b**) Calibration fit residuals: all the points are within ±0.6 mK.

Both fractional frequency change and *Q* factor are inverse functions of the temperature with a sensitivity depending on the selected whispering gallery mode. For instance, for WGM_n=6_ (14 GHz) the fractional frequency change varies from −65 × 10^−6^ at 0 °C to −62 × 10^−6^ at −40 °C. These values are in accordance to that reported in the literature [9]. The highest *Q* factor is reached at 12 GHz by the WGM_n=5_; its value is 173,000 at 0 °C and becomes 257,000 at −40 °C. Due to such a high *Q* factor and temperature sensitivity, the mode WGM_n=5_ at 12 GHz was selected for the operation of SWGT. The temperature calibration was then carried out in the whole temperature range and the experimental points (resonance frequency *f*_0_ vs. temperature) were fitted using a 5th order polynomial [11]. The calibration curve and the fitting residuals are shown in Figure 10a,b, respectively. A weighted fit, carried out using the inverse of variance (1/σ^2^) as weight, resulted in fitting residuals of better than ±0.6 mK in the investigated temperature range.

**Figure 11:**
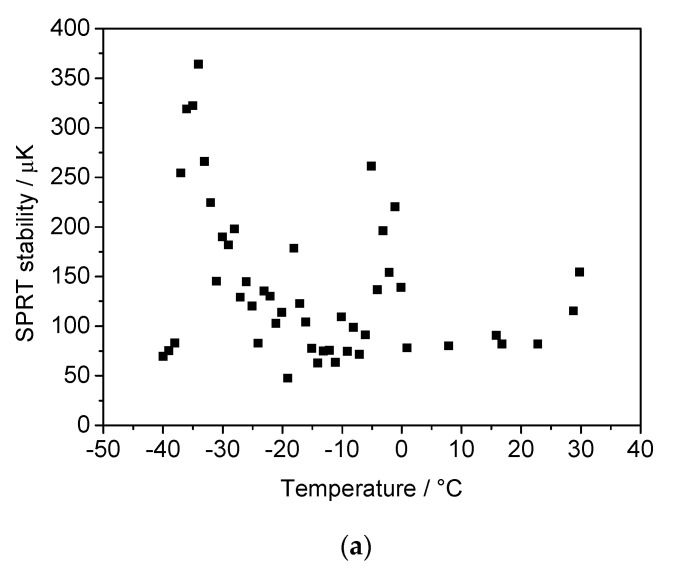

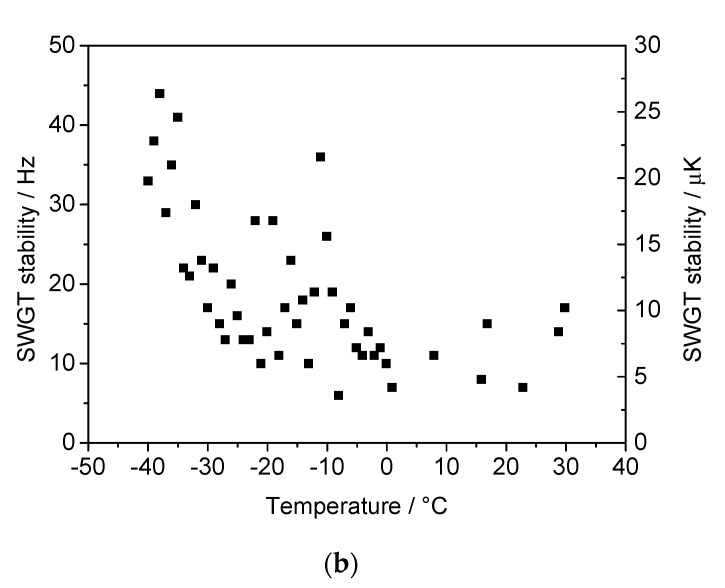
(**a**) Stability of the temperature calibration bath in terms of standard deviation at each temperature set point. (**b**) Stability of the resonant frequency in terms of standard deviation expressed in Hz and converted in temperature unit. The SWGT temperature measurement is more stable compared to the bath temperature because of the large copper cavity thermal mass, which was able to filter out the bath temperature fluctuations.

The temperature stability at each calibration point was also investigated both in the thermostatic calibration bath and in the copper cavity, which hosts the SWGT. For each temperature set point, the PID controller parameters of the bath were set for the best performances. The calibration bath stability was estimated in terms of the SPRT standard deviation and was always lower than 400 mK (Figure 11a). Although it was limited with respect to the potential SWGT performance, the actual temperature fluctuations in the copper cavity were smoothed down due to the high thermal mass of the resonator cavity. A standard deviation lower than 45 Hz was observed at all SWGT resonance frequency measurements. Considering an average sensitivity of −60 ppb/mK, this would correspond to a worst-case temperature stability of 30 µK with an average stability of 15 µK (Figure 11b).

### 3.2. Stability and Repeatability at the Ice Melting Point

**Figure 12:**
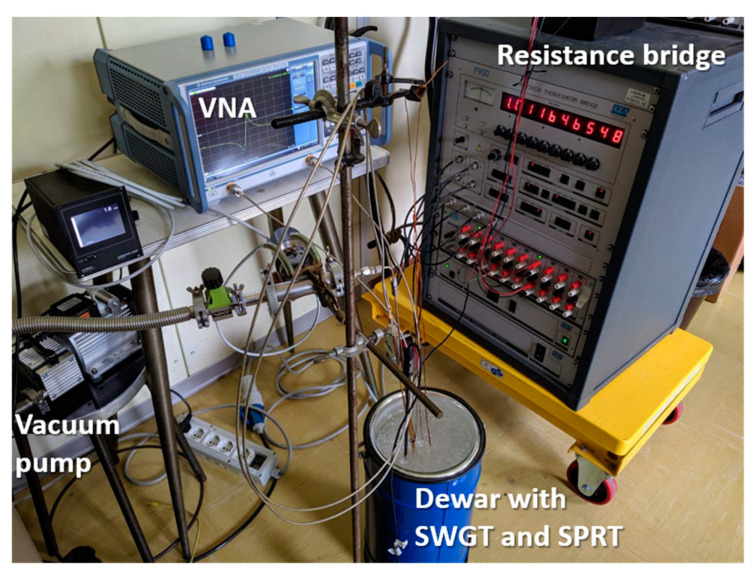
Measurement setup for the ice melting point stability test: SWGT inside the Dewar flask with the SPRT. They are connected to the VNA and the resistance bridge, respectively.

**Figure 13:**
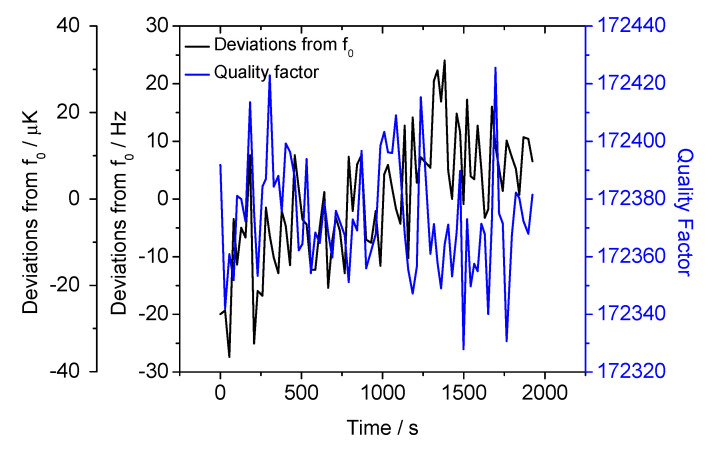
SWGT resonance frequency and *Q* factor deviations when the microwave resonator is placed inside a melting ice bath.

The measurement stability and repeatability of the SWGT was also evaluated at a temperature fixed point by placing the resonator into a melting ice bath. The measurement setup was the same as described before; a Dewar flask with melting ice (Figure 12) replaced the thermostatic bath, improving the reference temperature stability. A 30-min observation of the SWGT resonance frequency showed a peak-to-peak fluctuation of ±30 Hz with an estimated standard deviation of 11 Hz. This would correspond to a standard deviation of 14 μK in the temperature unit (Figure 13).

It is worth comparing the SWGT measurement stability with the SPRT temperature stability, since they were both hosted in the same copper cavity and were in thermal equilibrium during the whole experiment. The temperature fluctuations detected by the SPRT were thus converted into equivalent resonant frequency deviations by using the SWGT sensitivity (*S_SWGT_*).

(7) $$\Delta f=\Delta T_{SPRT}\cdot S_{SWGT}$$

**Figure 14:**
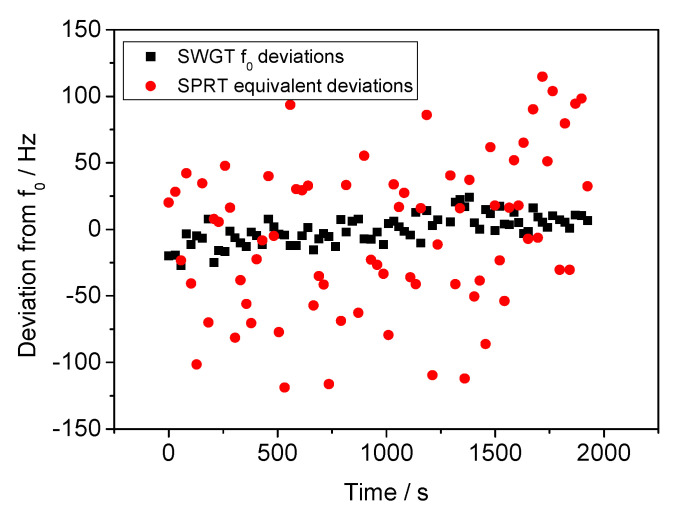
Comparison between the measurement stability of SWGT (black squares) and SPRT (red circles). In this plot the SPRT resistance deviations have been converted into equivalent frequency deviations. The SWGT measurement stability is about five times better than the SPRT.

The SWGT measurement stability was about five times better than the equivalent SPRT stability. This comparison is shown in Figure 14. This result is in line with that reported in the literature [13] for a SWGT and confirms the good performance of this device for temperature measurements.

**Figure 15:**
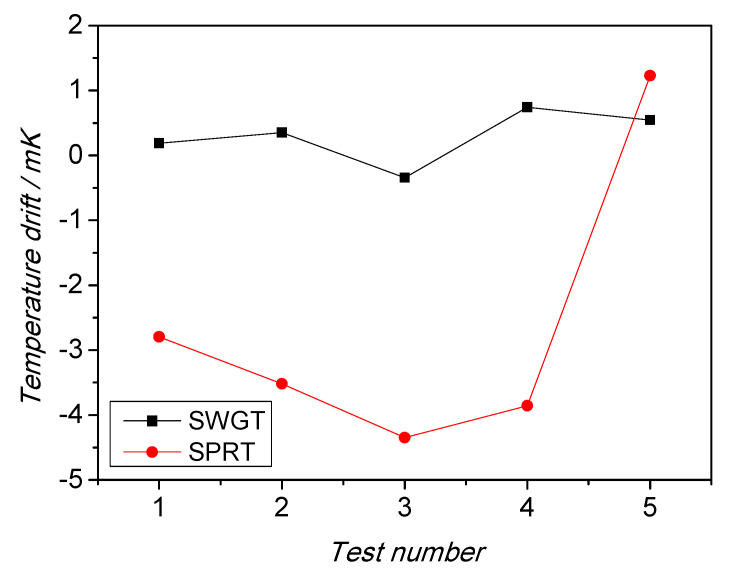
Comparison between the measurement repeatability of SWGT (black squares) and SPRT (red circles). Additionally, in this case the SWGT performs better.

Beside stability, the SWGT repeatability at the ice melting point was evaluated, as well. Measurements were repeated several times with different realizations of the ice point, each time recording the temperature from SPRT and the equivalent temperature from SWGT. Additionally, in this case the performance of the microwave thermometer exceeded that of the SPRT. Figure 15 shows the deviation of successive measurement from the initial one, took as a reference. The SWGT measurements showed a drift consistently below 1 mK, while the SPRT drift was up to 5 mK.

The overall results highlighted the performance of the microwave-based SWGT in comparison to the industry-standard platinum resistance thermometer, confirming what was claimed by Strouse [9] in its seminal paper that “SWGT represents a potential replacement for an SPRT in industrial applications where measurement uncertainty below 10 mK is required”.

### 3.3. Uncertainty Budget Analysis

The measurement uncertainty of this microwave-based SWGT was estimated by considering the main uncertainty components as reported in Table 2 and Table 3.

Considering all the contributions reported in Table 2, the combined standard measurement uncertainty (*k* = 1) of the SWGT calibration was estimated to be 1.2 mK, approximately three times better than that achieved in previous work [11]. The contributions coming from the SWGT itself, highlighted in grey area in Table 2, were very low (<450 μK). The higher uncertainty contribution came from the calibration process, in particular from temperature bath performance. By improving the calibration process, it would be possible to further reduce the overall SWGT measurement uncertainty.

## 4. Discussion and Conclusions

The paper reported the design, development and testing of a sapphire whispering gallery-mode thermometer (SWGT). This work is based and further progressed the work by Strouse [9] in the use of a cylindrical sapphire as the dielectric resonator similar and Yu’s studies in cavity design [18,19]. However, a significant improvement was achieved by redesigning the copper cavity in order to improve the resonator mechanical stability at the lower temperatures. Due to the new cavity design, a better mechanical stability was also observed over the whole temperature range, by greatly reducing the impact of temperature-related sapphire displacement and tilting effects. Moreover, the vacuum sealing was improved.

A thoroughly investigation was carried out to validate and confirm the performance of the SWGT in a temperature range from −40 to 30 °C. A very high measurement stability (σ = 14 µK) was recorded at the ice melting point temperature. This improvement respect to the previous work was possible by means of the selected sapphire resonator. Its bigger size ensures a higher *Q* factor so that the SWGT frequency resolution increases [8]. The bigger cavity thermal mass certainly slowed down the sensor response time, but it increased considerably the sapphire temperature stability improving the SWGT performances. The measurement results showed that the calibration standard measurement uncertainty (*k* = 1) was 1.2 mK.

The main limitation to a full investigation of the SGWT performance came from the calibration bath. This contribution can be improved in future measurements with a better temperature control system or with the use of temperature fixed points. Further activities will be focused on the evaluation of the sensor interchangeability and system reproducibility by considering different prototypes of SWGTs. 

One of the possible applications for the SWGT is as a transfer standard in industrial applications to replace the industry-standard PRT. The SWGT could be a more robust alternative to SPRTs and industrial PRTs in some areas like aerospace and chemical industry where a measurement uncertainty below 10 mK is often needed. Furthermore, because of its high measurement resolution, the SWGT can also be exploited in investigations of SPRT non-linearity and its resistivity variation with temperature. SPRTs, calibrated at fixed points, are used for the dissemination of the temperature unit via the International Temperature Scale of 1990 (ITS-90). The SPRT resistance is well characterized at any temperature fixed point, which approximates the thermodynamic temperature *T*, but less characterized between consecutive fixed points with known deviations between the SPRT interpolating equation (*T*(*R_SPRT_*)) and its real electrical resistance [27,28]. Such deviations, known as ITS-90 non-uniqueness *T-T*_90_, need to be better elucidated and the SWGT could be a suitable instrument in this regard.

## Figures and Tables

**Table 1:** Whispering gallery modes with *n* = 2 to *n* = 6 (*l* = 1, *m* = *n*). Simulated (sim.) versus measured (meas.) resonant frequency (*f*) and Q factor.

WGM	*f*/GHz (sim./Meas.)	*Q* (sim./Meas.)
*n* = 2	6.580/6.581	88,703/39,690
*n* = 3	8.482/8.487	120,890/111,700
*n* = 4	10.375/10.383	141,020/158,276
*n* = 5	12.263/12.251	152,130/173,442
*n* = 6	14.121/14.105	151,945/164,025

**Table 2:** Individual uncertainty components of the SWGT calibration. The estimates under a gray area refer to the temperature uncertainty contributions directly attributed to SWGT sensing capability.

Uncertainty Component	Estimate/μK
Frequency measurement ^1^	26
SWGT measurement stability	14
SWGT measurement repeatability	400
SWGT calibration curve fitting	200
Thermostatic bath stability	400
Thermostatic bath uniformity	1000
SPRT calibration	300
Thermometry bridge linearity	10

^1^ The uncertainty contributions due to the frequency measurements are listed in Table 3.

**Table 3:** Individual temperature uncertainty components due to frequency measurements.

Uncertainty Component	Estimate/μK
Lorentzian fitting reproducibility	10
VNA accuracy	5
10 MHz frequency reference stability	23
VNA resolution	5

## Data Availability

Not applicable.
